# COVID-19 Vaccine Effectiveness in Autumn and Winter 2022 to 2023 Among Older Europeans

**DOI:** 10.1001/jamanetworkopen.2024.19258

**Published:** 2024-07-01

**Authors:** Charlotte Laniece Delaunay, Clara Mazagatos, Iván Martínez-Baz, Gergő Túri, Luise Goerlitz, Lisa Domegan, Adam Meijer, Ana Paula Rodrigues, Noémie Sève, Maja Ilić, Neus Latorre-Margalef, Mihaela Lazar, Marine Maurel, Aryse Melo, Blanca Andreu Ivorra, Itziar Casado, Judit Krisztina Horváth, Silke Buda, Charlene Bennett, Marit de Lange, Raquel Guiomar, Vincent Enouf, Ivan Mlinarić, Tove Samuelsson Hagey, Sorin Dinu, Mercedes Rumayor, Jesús Castilla, Beatrix Oroszi, Ralf Dürrwald, Joan O’Donnell, Mariëtte Hooiveld, Verónica Gomez, Alessandra Falchi, Sanja Kurečić Filipović, Lena Dillner, Rodica Popescu, Sabrina Bacci, Marlena Kaczmarek, Esther Kissling

**Affiliations:** 1 Epidemiology Department, Epiconcept, Paris, France; 2 National Centre for Epidemiology, Institute of Health Carlos III, Madrid, Spain; 3 Consortium for Biomedical Research in Epidemiology and Public Health (CIBERESP), Madrid, Spain; 4 Instituto de Salud Pública de Navarra–IdiSNA, Pamplona, Spain; 5 National Laboratory for Health Security, Epidemiology and Surveillance Centre, Semmelweis University, Budapest, Hungary; 6 Department for Infectious Disease Epidemiology, Unit 36 Respiratory Infections, Robert Koch Institute, Berlin, Germany; 7 Health Service Executive-Health Protection Surveillance Centre, Dublin, Ireland; 8 Centre for Infectious Diseases Control, National Institute for Public Health and the Environment, Bilthoven, the Netherlands; 9 Epidemiology Department, National Institute of Health Doutor Ricardo Jorge, Lisbon, Portugal; 10 Sorbonne Université, Institut National de la Santé et de la Recherche Médicale (INSERM), Institut Pierre Louis d'épidémiologie et de Santé Publique (IPLESP UMRS 1136), Paris, France; 11 Division for Epidemiology of Communicable Diseases, Croatian Institute of Public Health, Zagreb, Croatia; 12 Department of Microbiology, The Public Health Agency of Sweden, Stockholm, Sweden; 13 National Influenza Centre, “Cantacuzino” National Military-Medical Institute for Research and Development, Bucharest, Romania; 14 Reference Laboratory for Influenza and Other Respiratory Virus, National Institute of Health Doutor Ricardo Jorge, Lisbon, Portugal; 15 Servicio de Epidemiología, Sección de Vigilancia Epidemiológica, Consejería de Salud de Murcia, Murcia, Spain; 16 National Virus Reference Laboratory, University College Dublin, Dublin, Ireland; 17 Institut Pasteur, Centre National de Référence Virus des Infections Respiratoires (CNR VIR), Paris, France; 18 Área de Enfermedades Transmisibles, Subdirección General de Vigilancia en Salud Pública, Madrid, Spain; 19 Department of Infectious Diseases, Unit 17 Influenza and Other Respiratory Viruses, Robert Koch Institute, Berlin, Germany; 20 Nivel (Netherlands Institute for Health Services Research), Utrecht, the Netherlands; 21 Laboratoire de Virologie, UR7310 Campus Grimaldi, Université de Corse, Corte, France; 22 National Center for Communicable Diseases Surveillance and Control, National Institute of Public Health, Bucharest, Romania; 23 European Centre for Disease Prevention and Control, Stockholm, Sweden

## Abstract

**Importance:**

In the context of emerging SARS-CoV-2 variants or lineages and new vaccines, it is key to accurately monitor COVID-19 vaccine effectiveness (CVE) to inform vaccination campaigns.

**Objective:**

To estimate the effectiveness of COVID-19 vaccines administered in autumn and winter 2022 to 2023 against symptomatic SARS-CoV-2 infection (with all circulating viruses and XBB lineage in particular) among people aged 60 years or older in Europe, and to compare different CVE approaches across the exposed and reference groups used.

**Design, Setting, and Participants:**

This case-control study obtained data from VEBIS (Vaccine Effectiveness, Burden and Impact Studies), a multicenter study that collects COVID-19 and influenza data from 11 European sites: Croatia; France; Germany; Hungary; Ireland; Portugal; the Netherlands; Romania; Spain, national; Spain, Navarre region; and Sweden. Participants were primary care patients aged 60 years or older with acute respiratory infection symptoms who were recruited at the 11 sites after the start of the COVID-19 vaccination campaign from September 2022 to August 2023. Cases and controls were defined as patients with positive and negative, respectively, reverse transcription–polymerase chain reaction (RT-PCR) test results.

**Exposures:**

The exposure was COVID-19 vaccination. The exposure group consisted of patients who received a COVID-19 vaccine during the autumn and winter 2022 to 2023 vaccination campaign and 14 days or more before symptom onset. Reference group included patients who were not vaccinated during or in the 6 months before the 2022 to 2023 campaign (seasonal CVE), those who were never vaccinated (absolute CVE), and those who were vaccinated with at least the primary series 6 months or more before the campaign (relative CVE). For relative CVE of second boosters, patients receiving their second booster during the campaign were compared with those receiving 1 booster 6 months or more before the campaign.

**Main Outcomes and Measures:**

The outcome was RT-PCR–confirmed, medically attended, symptomatic SARS-CoV-2 infection. Four CVE estimates were generated: seasonal, absolute, relative, and relative of second boosters. CVE was estimated using logistic regression, adjusting for study site, symptom onset date, age, chronic condition, and sex.

**Results:**

A total of 9308 primary care patients were included, with 1687 cases (1035 females; median [IQR] age, 71 [65-79] years) and 7621 controls (4619 females [61%]; median [IQR] age, 71 [65-78] years). Within 14 to 89 days after vaccination, seasonal CVE was 29% (95% CI, 14%-42%), absolute CVE was 39% (95% CI, 6%-60%), relative CVE was 31% (95% CI, 15% to 44%), and relative CVE of second boosters was 34% (95% CI, 18%-47%) against all SARS-CoV-2 variants. In the same interval, seasonal CVE was 44% (95% CI, −10% to 75%), absolute CVE was 52% (95% CI, −23% to 82%), relative CVE was 47% (95% CI, −8% to 77%), and relative CVE of second boosters was 46% (95% CI, −13% to 77%) during a period of high XBB circulation. Estimates decreased with time since vaccination, with no protection from 180 days after vaccination.

**Conclusions and Relevance:**

In this case-control study among older Europeans, all CVE approaches suggested that COVID-19 vaccines administered in autumn and winter 2022 to 2023 offered at least 3 months of protection against symptomatic, medically attended, laboratory-confirmed SARS-CoV-2 infection. The effectiveness of new COVID-19 vaccines against emerging SARS-CoV-2 variants should be continually monitored using CVE seasonal approaches.

## Key Points

**Question:**

What was the effectiveness of COVID-19 vaccines administered in autumn and winter 2022 to 2023 against symptomatic SARS-CoV-2 infection among people aged 60 years or older in Europe, and how did different exposed or reference groups affect effectiveness?

**Findings:**

In this case-control study of 9308 primary care patients at 11 European sites, within 3 months of vaccination, all COVID-19 vaccine effectiveness (CVE) estimates were 29% to 39% against SARS-CoV-2 viruses and 44% to 52% against the XBB variants. All point estimates decreased by time after vaccination, with no vaccine protection after 6 months.

**Meaning:**

Findings of this study suggest that COVID-19 vaccination campaigns should precede peaks in SARS-CoV-2 incidence and that effectiveness of new vaccines against emerging variants should be continually monitored using seasonal CVE approaches.

## Teaser

This case-control study estimates the effectiveness of COVID-19 vaccines for patients 60 years or older with varying vaccination histories during the autumn and winter 2022 to 2023 vaccination campaign.

## Introduction

In Europe, SARS-CoV-2 viruses circulating between September 2022 and August 2023 were successively dominated by different Omicron lineages: BA.5, BQ.1, and XBB (including the XBB.1.5 and XBB.1.5 + F456L sublineages).^1^ Bivalent messenger RNA (mRNA) vaccines containing original and Omicron BA.1 components and original and BA.4-5 or BA.5 components were introduced in the European Union (EU) and European Economic Area in autumn 2022.^2^ These vaccines were used as boosters in the autumn and winter 2022 to 2023 COVID-19 vaccination campaigns that targeted adults aged 60 years or older (age cutoffs varied across countries).^3,4^ Emerging evidence suggests that mRNA bivalent boosters protected against symptomatic infection with BA.5 and XBB lineages.^5^ While in autumn 2023 the XBB.1.5 monovalent vaccine was introduced and widely used in Europe, the effectiveness of bivalent BA.4-5 or BA.5 vaccines remains relevant in understanding the magnitude and duration of COVID-19 vaccine effectiveness (CVE).^6^

Methods for CVE estimation have evolved since COVID-19 vaccines were first introduced, with different exposed and reference groups.^7,8^ Absolute CVE using never-vaccinated people as a reference now poses several challenges. First, most people targeted by COVID-19 vaccination campaigns have been vaccinated; in September 2022, over 90% of adults aged 60 years or older had received a primary series vaccination in the EU and European Economic Area.^9^ Using a reference group comprising only never-vaccinated people may therefore lead to a small sample size and/or generalizability issues. Second, it is increasingly difficult to collect complete COVID-19 vaccination history (ie, date, vaccine brand, and composition of each dose). In many studies, exposure can no longer be defined as a precise dose number. Third, because most target populations have been vaccinated, the CVE estimate of highest public health interest is the protection conferred by 1 additional dose of vaccine (with an adapted vaccine if relevant) regardless of vaccination history.

In Europe, SARS-CoV-2 circulation now follows more seasonal patterns,^10^ and countries organize annual or biannual COVID-19 vaccination campaigns.^3,4^ These evolutions have incited researchers to transition into an influenza model to estimate CVE, in which people vaccinated during a campaign are compared with people not vaccinated during that campaign.^11^ In this model, the number of doses received is not considered, and CVE reflects vaccine protection conferred to a population with heterogeneous history of vaccination and SARS-CoV-2 infection. Nevertheless, SARS-CoV-2 is still a novel virus in constant mutation. The implications for CVE of SARS-CoV-2 mutations and the cross-reactivity of immunity raised by past infections are not fully understood.^12,13,14^ Vaccines and methodological approaches to CVE are also evolving rapidly. Alongside the influenza model, different CVE analyses focusing on various exposure and reference groups allow us to answer different public health questions. Therefore, it is critical to understand how CVE estimates vary with changes in exposure and reference group definitions within the same study period and population.

In this study, we aimed to answer 2 research questions. First, how well did COVID-19 vaccines administered during the autumn and winter 2022 to 2023 vaccination campaigns protect against medically attended, laboratory-confirmed symptomatic SARS-CoV-2 infection (with all circulating SARS-CoV-2 viruses and XBB lineage in particular) among people aged 60 years or older from September 2022 to August 2023 in Europe? Second, how did these CVE estimates vary among the exposed and reference groups used?

## Methods

### Study Design and Population

The Vaccine Effectiveness, Burden and Impact Studies (VEBIS) of COVID-19 and influenza, funded by the European Centre for Disease Prevention and Control, is a multicenter case-control study that collects data from 11 European sites: Croatia; France; Germany; Hungary; Ireland; Portugal; the Netherlands; Romania; Spain, national; Spain, Navarre region; and Sweden.^15^ The VEBIS primary care network has generated estimates of COVID-19^16^ and influenza^17^ vaccine effectiveness, and these VEBIS data were used in the present case-control study. All sites participating in the VEBIS primary care network also participated in the Influenza-Monitoring Vaccine Effectiveness in Europe (I-MOVE) primary care network, which estimated influenza vaccine effectiveness between 2008 and 2022^18^; most sites participated in the I-MOVE-COVID-19 network, estimating CVE in 2021.^7,19^ Ethical approval was not required in Spain or the Netherlands because this study was classified as routine care or surveillance. A national or regional review board in other countries provided ethical approval in Croatia, France, Germany, Hungary, Ireland, Portugal, Romania, and Sweden. Patient consent was not required in Ireland or Spain, and verbal informed consent was obtained in all other countries, except in Germany and Hungary, where written informed consent was required and obtained. We followed the Strengthening the Reporting of Observational Studies in Epidemiology (STROBE) reporting guideline.

In each site, we selected all or a systematic sample of patients who consulted primary care physicians with symptoms meeting the EU’s acute respiratory infection definition (sudden onset of symptoms; at least 1 respiratory symptom: cough, sore throat, shortness of breath, and coryza; and a clinician’s judgment that the illness is due to an infection).^16,20^ Site-specific variations in case definitions are described in eTable 1 in Supplement 1. Physicians swab participants, and COVID-19 case or control status is defined by a positive or negative SARS-CoV-2 reverse transcription–polymerase chain reaction (RT-PCR) test result, respectively. Demographic and clinical data, including age, sex, and information on symptoms and chronic conditions, are collected via an interview and/or via linkage to or consultation of electronic medical records. COVID-19 vaccination information is self-reported or obtained from national registries; the sources are listed by site in eTable 1 in Supplement 1.

We excluded patients for whom COVID-19 vaccination was contraindicated; those who lived in a residential care facility; and patients who had missing or inconsistent demographics data (including age), dates (symptom onset, specimen collection, and most recent COVID-19 vaccination), case status (inconclusive or no SARS-CoV-2 test result, no test type, or antigen test only), or COVID-19 vaccination (received vaccines not approved by the European Medicines Agency or unknown).

### Study Period

To estimate CVE against infection with any SARS-CoV-2 virus circulating in the 2022 to 2023 season, we included patients swabbed 14 days or more after the start of the autumn and winter 2022 to 2023 vaccination campaign (up to August 31, 2023) in each country. To estimate CVE against infection with XBB, we used data from the Global Initiative on Sharing All Influenza Data or The European Surveillance System.^1,21^ Using genetic characterization data available from these sources, we identified the weeks when XBB (including the XBB.1.5 and XBB.1.5 + F456L sublineages) represented over 60% of SARS-CoV-2 viruses sequenced in each country and included study patients who were swabbed during those weeks only.

### Outcome, Exposures, and Reference Groups

For all analyses, the outcome was RT-PCR–confirmed, medically attended, symptomatic SARS-CoV-2 infection. The exposure was COVID-19 vaccination. We generated 4 CVE estimates (Table 1). In all exposure definitions, patients were considered to be vaccinated in the autumn and winter 2022 to 2023 campaign if they received the vaccine of interest after the start of the country-specific vaccination campaign and 14 days or more before symptom onset. In all reference group definitions, patients were considered to be not vaccinated during the autumn and winter 2022 to 2023 campaign if they received their last dose 6 months or more before that campaign (the precampaign cutoff) or if they were never vaccinated.

**Table 1.** Definitions of the Exposed and Reference Groups for Different COVID-19 Vaccine Effectiveness (CVE) Estimates

CVE estimate	Exposed group	Reference group
Seasonal CVE	Vaccinated with any COVID-19 vaccine in autumn or winter 2022-2023	Not vaccinated in autumn or winter 2022-2023 or in 6 mo preceding that vaccination campaign
Absolute CVE		Never vaccinated against COVID-19
Relative CVE		Vaccinated with at least a full primary series but not in autumn or winter 2022-2023 or in 6 mo preceding that vaccination campaign
Relative CVE (second booster)	Received second COVID-19 vaccination booster in autumn or winter 2022-2023, but no additional dose	Fully vaccinated with primary series plus 1 booster dose (only); not vaccinated in autumn or winter 2022-2023 or in 6 mo preceding that vaccination campaign

For seasonal CVE, we compared patients who received any COVID-19 vaccine during the campaign (exposed group) with patients who were not vaccinated during the campaign (reference group). For absolute CVE, we compared patients who received any COVID-19 vaccine during the campaign (exposed group) with patients who were never vaccinated (reference group). For relative CVE, we compared patients who received any COVID-19 vaccine during the campaign (exposed group) with patients who received at least a complete primary series before the precampaign cutoff. For relative CVE of second boosters, we compared patients who received their second booster during the campaign and no additional dose (exposed group) with patients who received a first booster and no additional dose before the precampaign cutoff (reference group) (Table 1).

### CVE Analyses

In descriptive analyses, we described the number of cases and controls recruited by week of swab in the study period for CVE against infection with any SARS-CoV-2 virus and during a period of high XBB circulation specifically. We also described baseline characteristics of cases and controls.

We excluded sites with fewer than 10 cases or controls from each CVE analysis. We estimated seasonal, absolute, relative, and relative CVE of second boosters against infection with any SARS-CoV-2 virus circulating in the 2022 to 2023 season, and during the period of high XBB circulation specifically, by time since vaccination. We performed complete case analyses, excluding patients with missing information on any model covariate. We estimated CVE with logistic regression models using the following formula:

CVE = 1 − (odds of vaccination in cases)/(odds of vaccination in controls) × 100.

We adjusted for the following a priori confounders: study site; symptom onset date; age; sex; and presence of at least 1 of the following commonly collected chronic conditions: diabetes, immunodeficiency, lung disease, and heart disease. We modeled study site as a categorical variable and chronic condition and sex as binary variables. We explored various functional forms for age and symptom onset date (continuous, onset month, age in 5-year bands up to 75 years, and restricted cubic splines using the location of knots as specified by Harrell^22^ and varying between 3 and 5 knots). We chose the best-fitting model among fully adjusted models, using the Akaike information criterion.^23^ We also examined the magnitude of regression coefficients and their SEs to check for high SEs compared with coefficients (unstable results).

To minimize bias associated with overfitted models, we repeated CVE analyses using penalized regression described by Firth^24^ if there were fewer than 10 events per model parameter, fewer than 20 exposed or unexposed patients, fewer than 10 cases or controls, or fewer than 5 events per cell of the table formed by the exposure and outcome variables.^25,26,27^ If the penalized regression estimate differed by 10% or more from the standard logistic regression estimate, we did not report that estimate.

In sensitivity analyses, we varied the definition of the precampaign cutoff for the reference group. We used 3 months prior to the start of the vaccination campaign (instead of 6 months, as used in the main analyses) as well as 3 and 6 months prior to symptom onset. These sensitivity analyses did not apply to absolute CVE, as this reference group comprised only patients who were never vaccinated.

### Statistical Analysis

All analyses were performed in R version 4.3.2 (R Project for Statistical Computing). Additional R packages for analysis included logistf() for penalized logistic regression and rms() for restricted cubic splines.

## Results

### Descriptive Analyses

**Figure 1.**
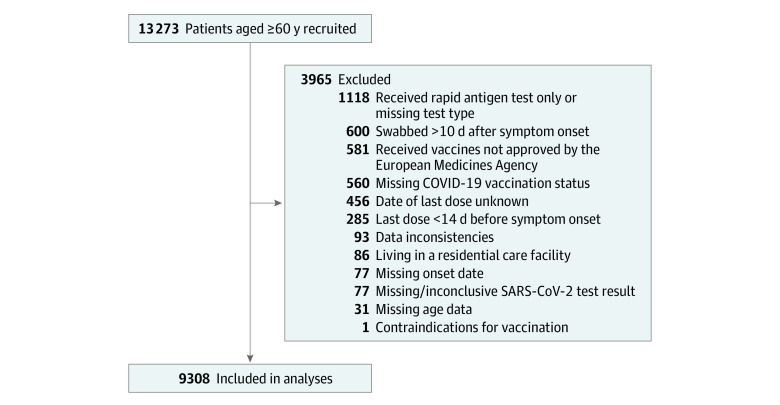
Study Restriction Flowchart

**Figure 2.**
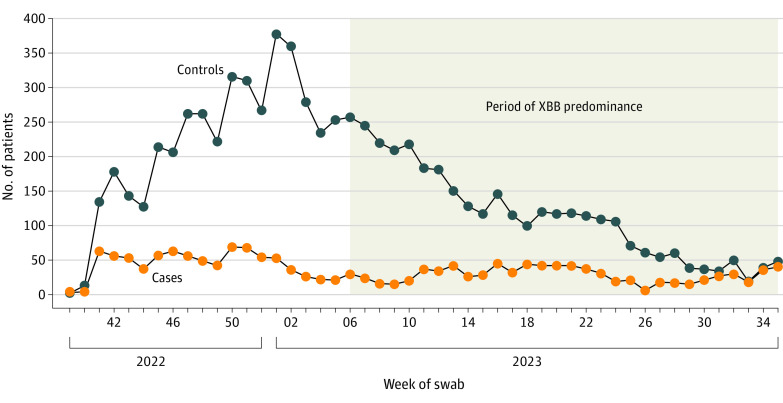
COVID-19 Cases and Test-Negative Controls Recruited by Week of Swab From September 2022 to August 2023 Controls were individuals with a negative SARS-CoV-2 reverse transcription–polymerase chain reaction test result. The period of XBB predominance varied across countries in the VEBIS (Vaccine Effectiveness, Burden and Impact Studies) Primary Care Vaccine Effectiveness Group. This period spanned from the first week of XBB predominance in the first country to the last week of XBB predominance in the last country.

We recruited 13 273 patients between the start of country-specific COVID-19 vaccination campaigns in September to November 2022 (eTable 2 in Supplement 1) and August 31, 2023 (Figure 1). We excluded 3965 of these patients. We included 1687 COVID-19 cases (1035 females [61%], 651 males [39%]; median [IQR] age, 71 [65-79] years) and 7621 test-negative controls (4619 females [61%], 2995 males [39%]; median [IQR] age, 71 [65-78] years) in the analyses (Figure 2; Table 2). Depending on the country, XBB dominated from February, March, or April 2023 until August 31, 2023.

**Table 2.** Baseline Characteristics of Adults 60 Years or Older in the Vaccine Effectiveness, Burden and Impact Studies

Characteristic	No. (%)^a^	
	COVID-19 cases (n = 1687)	Test-negative controls (n = 7621)^b^
Age, median (IQR), y	71 (65-79)	71 (65-78)
Age group, y		
60-64	393 (23)	1825 (24)
65-69	345 (20)	1598 (21)
70-74	260 (15)	1426 (19)
≥75	689 (41)	2772 (36)
Missing data	0	0
Sex		
Female	1035 (61)	4619 (61)
Male	651 (39)	2995 (39)
Missing data	1	7
Chronic condition^c^		
With chronic condition	1100 (69)	5043 (69)
Without chronic condition	494 (31)	2222 (31)
Missing data	93	356
Vaccinated in the autumn or winter 2022-2023		
Yes	645 (38)	3248 (43)
No	1042 (62)	4373 (57)
Missing data	0	0
Last dose received in autumn or winter 2022-2023		
First dose of a 2-dose primary series	0	4 (<1)
Last dose of primary series	3 (<1)	6 (<1)
First booster	17 (3)	112 (3)
Second booster	594 (92)	2955 (91)
Third booster	31 (5)	167 (5)
Fourth booster	0	4 (<1)
Missing data	0	0
Study site		
Croatia	46 (3)	45 (1)
France	31 (2)	110 (1)
Germany	35 (2)	289 (4)
Hungary	89 (5)	328 (4)
Ireland	34 (2)	258 (3)
The Netherlands	33 (2)	207 (3)
Portugal	19 (1)	147 (2)
Romania	2 (<1)	23 (<1)
Sweden	15 (1)	56 (1)
Spain, national	1205 (71)	5516 (72)
Spain, Navarra region	178 (11)	642 (8)
Missing data	0	0

^a^ Percentages were calculated without missing data.

^b^ Controls were individuals with negative SARS-CoV-2 reverse transcription–polymerase chain reaction test result.

^c^ Presence of at least 1 of the following commonly collected chronic conditions: diabetes, immunodeficiency, lung disease, and heart disease.

Sixty-nine percent of cases (1100 of 1594) and controls (5043 of 7265) lived with a chronic condition. Thirty-eight percent of cases (645 of 1687) and 43% of controls (3248 of 7621) received a COVID-19 vaccine in autumn or winter 2022 to 2023. This vaccine was a second booster for 92% of cases (594 of 645) and 91% of controls (2955 of 3248) (Table 2).

### COVID-19 Vaccine Effectiveness

**Figure 3.**
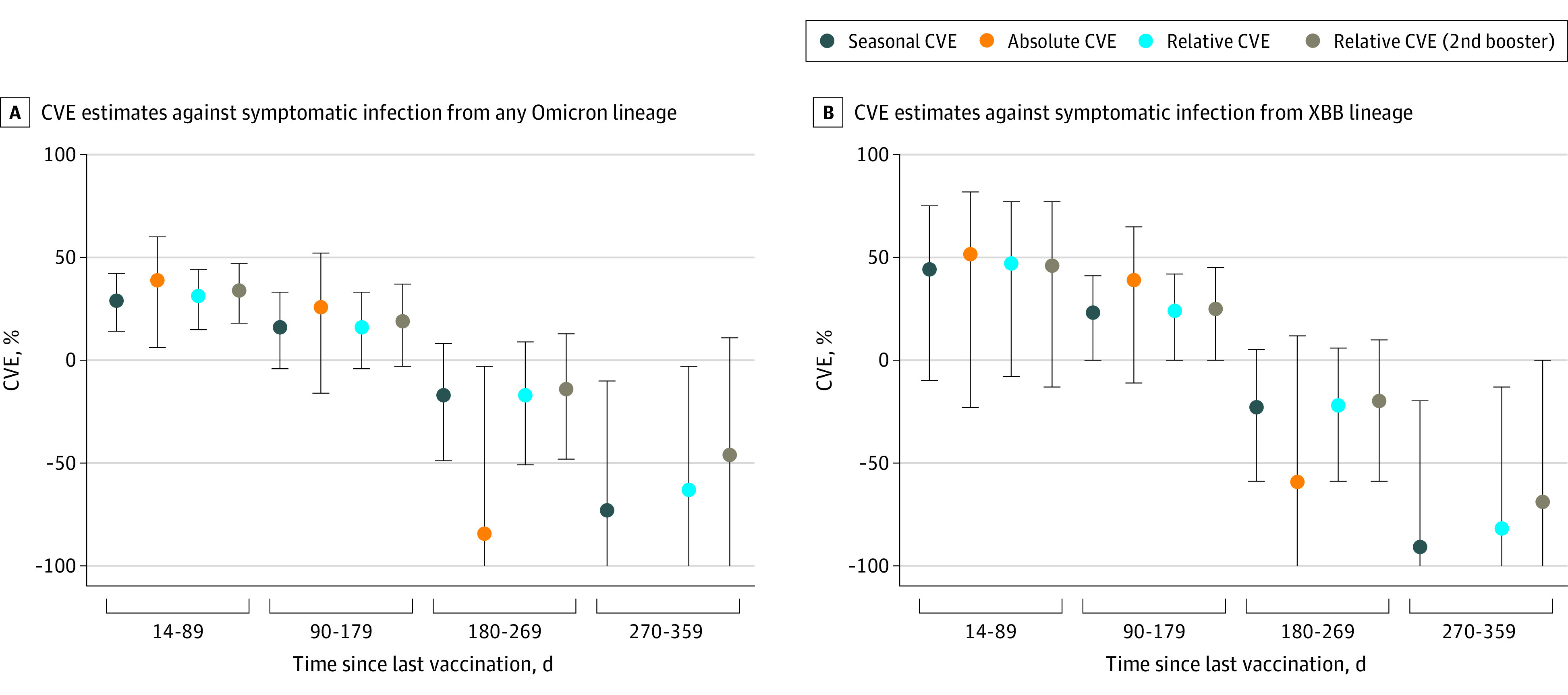
COVID-19 Vaccine Effectiveness (CVE) in Autumn and Winter 2022 to 2023 Against SARS-CoV-2 Variants Circulating Between September 2022 and August 2023 Among Adults 60 Years or Older Error bars represent 95% CIs. The y-axis is truncated at −100; eTable 3 in Supplement 1 provides the exact numbers for all confidence limits.

Seasonal CVE against infection with any SARS-CoV-2 virus was 29% (95% CI, 14%-42%) in the 14 to 89 days after vaccination, 16% (95% CI, −4% to 33%) at 90 to 179 days, −17% (95% CI, −49% to 8%) at 180 to 269 days, and −73% (95% CI, −171% to −10%) at 270 to 359 days (Figure 3; eTable 3 in Supplement 1). Absolute CVE was 39% (95% CI, 6%-60%) in the 14 to 89 days after vaccination, 26% (95% CI, −16% to 52%) at 90 to 179 days, and −84% (95% CI, −245% to −3%) at 180 to 269 days. Absolute CVE estimates obtained using standard and penalized logistic regression for the 270- to 359-day interval differed by more than 10%. Relative CVE was 31% (95% CI, 15% to 44%) in the 14 to 89 days after vaccination, 16% (95% CI, −4% to 33%) at 90 to 179 days, −17% (95% CI, −51% to 9%) at 180 to 269 days, and −63% (95% CI, −160% to −3%) at 270 to 359 days. Relative CVE of second boosters was 34% (95% CI, 18%-47%) in the 14 to 89 days after vaccination, 19% (95% CI, −3% to 37%) at 90 to 179 days, −14% (95% CI, −48% to 13%) at 180 to 269 days, and −46% (95% CI, −141% to 11%) at 270 to 359 days.

Among patients swabbed during the XBB-dominant period, seasonal CVE was 44% (95% CI, −10% to 75%) in the 14 to 89 days after vaccination, 23% (95% CI, 0%-41%) at 90 to 179 days, −23% (95% CI, −59% to 5%) at 180 to 269 days, and −91% (95% CI, −207% to −20%) at 270 to 359 days (Figure 3; eTable 3 in Supplement 1). Absolute CVE was 52% (95% CI, −23% to 82%) in the 14 to 89 days after vaccination, 39% (95% CI, −11% to 65%) at 90 to 179 days, and −59% (95% CI, −199% to 12%) at 180 to 269 days. Absolute CVE estimates obtained using standard and penalized logistic regression for the 270- to 359-day interval differed by more than 10%. Relative CVE was 47% (95% CI, −8% to 77%) in the 14 to 89 days after vaccination, 24% (95% CI, 0% to 42%) at 90 to 179 days, −22% (95% CI, −59% to 6%) at 180 to 269 days, and −82% (95% CI, −196% to −13%) at 270 to 359 days. Relative CVE of second boosters was 46% (95% CI, −13% to 77%) in the 14 to 89 days after vaccination, 25% (95% CI, 0% to 45%) at 90 to 179 days, −20% (95% CI, −59% to 10%) at 180 to 269 days, and −69% (95% CI, −185% to 0%) at 270 to 359 days.

### Sensitivity Analyses

When changing the definition of the period without vaccination preceding the autumn and winter 2022 to 2023 vaccination campaign or the patient’s symptom onset date for the reference group, CVE estimates against any SARS-CoV-2 virus differed by 7% or less and those during the XBB-dominant period by 9% or less (eTables 4-6 in Supplement 1). Across analyses, between 0% and 8% of patients from the reference group were excluded due to vaccination in months preceding the campaign or the patient’s symptom onset date.

## Discussion

The effectiveness of COVID-19 vaccines administered in autumn and winter 2022 to 2023 ranged from 29% to 39% against any SARS-CoV-2 virus and from 44% to 52% against the XBB lineage within 89 days of vaccination. Effectiveness decreased by time since vaccination, with no protection from 180 days. Compared with relative or seasonal CVE, point estimates for absolute CVE were slightly higher within 179 days of vaccination and lower from 180 days. There was little difference between seasonal and relative CVE, and all CIs overlapped within time since vaccination periods.

In countries participating in VEBIS, mRNA bivalent vaccines were recommended as booster doses in autumn and winter 2022 to 2023.^3,4^ Although we did not have individual data on vaccine composition, site-specific study teams confirmed that bivalent vaccines represented the majority of boosters received. In this study, more than 99% of doses administered during the vaccination campaigns were boosters (Table 1). It is, therefore, reasonable to assume that the CVE estimates assessed the protection conferred by bivalent vaccines. This multicenter study thereby contributes to evidence on CVE of bivalent vaccines^5,8,28^ and can inform vaccination policies.

Comparing different CVE approaches within the same population and period can be challenging. We compared seasonal, absolute, and relative CVE within 1 population and period and by time since vaccination. This comparison allowed us to better understand the implications of each approach. In this setting, absolute CVE was comparable to other estimates. As vaccine-induced immunity decreases with time, people vaccinated 6 months or more before the vaccination campaign in the reference group may be similar in terms of protection to people who were never vaccinated. Thus, CVE could be estimated using just the last dose, without accounting for the number of doses received. In the VEBIS population, the never vaccinated and ever partially vaccinated groups were small, with the consequence that seasonal and relative CVE are likely to be similar, even if there were greater differences between absolute and relative CVE estimates.

We also explored changes in the date and period used for excluding recently vaccinated patients from the reference group. With a fixed date, such as 6 months before the start of a vaccination campaign, the time since vaccination among the reference group increases as the study progresses. With a relative date, such as each patient’s symptom onset date, as the study progresses the reference group includes the same patients as when using a fixed date but also includes patients who are vaccinated in the months before the vaccination campaign. Patients who are vaccinated just before a vaccination campaign may differ from patients who are vaccinated during vaccination campaigns in ways that are associated with vaccination and infection (eg, they may be more frail). One limitation of the fixed-date approach is that when considering an autumn and winter vaccination campaign, we may exclude people who are vaccinated during a possible spring vaccination campaign. Nevertheless, the sensitivity analyses showed a less than 10% absolute difference in CVE when varying these approaches.

We estimated particularly low (point estimates were below 0) CVE 9 to 12 months after vaccination. Differential depletion of COVID-19–susceptible persons among the exposed and reference groups could explain negative CVE estimates.^29^ Patients in the reference group have a longer time since vaccination than the exposed or unvaccinated group. Assuming the COVID-19 vaccine is protective, these patients are at higher risk for SARS-CoV-2 infection. Because infection protects against reinfection^30^ in a period of SARS-CoV-2 circulation, over time an increasing proportion of the reference group is at lower risk of becoming a case, biasing CVE downward. Without individual data on previous SARS-CoV-2 infection, ideally collected via regular testing, which was not possible due to the study design, we cannot correct for this potential bias. In this context, CVE estimates after a long time since vaccination (and, in this study, with a small sample size) should be carefully interpreted.

Link-Gelles et al^5^ estimated the relative CVE of mRNA bivalent boosters against symptomatic infection among adults aged 65 years or older in the US. Within 2 weeks to 3 months of vaccination, CVE was 37% against infection with BA.5 and 43% against infection with XBB. Tartof et al^28^ evaluated the CVE of bivalent second boosters against medically attended symptomatic infection with BA.4-5 or BA.5 or XBB in the US. Among adults aged 65 years or older, relative CVE was 27% within 0 to 3 months after vaccination and −18% within 4 to 7 months after vaccination. Absolute CVE was 29% within 0 to 3 months after vaccination and −15% within 4 to 7 months after vaccination. The magnitude of the estimates and the waning we observed in CVE are in line with results of other research.^5,28^

### Limitations

This study has several limitations. First, we lacked data on previous SARS-CoV-2 infection. Given that prior infection can confound and/or modify CVE, it would be beneficial to take this variable into account.^31,32^ Second, there was variation in source of COVID-19 vaccination information within VEBIS sites, with some sites using self-reported information. The largest proportion of patients in the study came from 1 country (Spain) due to the high coverage of their acute respiratory infection surveillance system. While there were no substantial differences between virus circulation and vaccines used between countries included in VEBIS, we cannot exclude population immunity differences between countries associated with public health as well as social measures and behavior. Therefore, if previous SARS-CoV-2 infection history in the population differed in Spain, this may affect the overall vaccine effectiveness due to its weight. Other heterogeneities within the data may exist that were associated with the vaccines received during a primary course vaccination and the distribution of patients by age and related differences in vaccine effectiveness due to immunosenescence. Small sample size did not allow further investigation into these factors. Third, a proxy measure was used for identifying XBB sublineages. Due to possible contamination of CVE estimates by the presence of other variants, differences between the overall SARS-CoV-2 virus analysis and XBB-specific results should be interpreted with caution.

## Conclusions

Within 6 months of vaccination, the COVID-19 vaccines administered in autumn and winter 2022 to 2023 offered some protection against symptomatic, medically attended, laboratory-confirmed SARS-CoV-2 infection among adults aged 60 years or older in Europe in a season of high levels of circulating Omicron viruses. The results of this study confirmed that a move away from absolute to seasonal CVE approaches can be valid. The effectiveness of new vaccine products against emerging variants should be continually monitored, including the mRNA XBB.1.5-specific vaccines authorized in autumn 2023.

## Notes

Supplement 1. **eTable 1.** Study Site-Specific COVID-19 Case Definition, VEBIS Primary Care Study, Europe, September 2022–August 2023 **eTable 2.** Start Date of the Autumn/Winter 2022/23 COVID-19 Vaccination Campaign by Study Site, VEBIS Primary Care Study, Europe, September 2022–August 2023 **eTable 3.** Effectiveness of COVID-19 Vaccines Administered in the Autumn/Winter 2022/23 Against Infection With SARS-CoV-2 Variants Circulating Between September 2022 and August 2023, Among Adults Aged 60 Years and Above, VEBIS Primary Care Study, Europe **eTable 4.** Effectiveness of COVID-19 Vaccines Administered in the Autumn/Winter 2022/23 Against Infection With SARS-CoV-2 Variants Circulating Between September 2022 and August 2023, Among Adults Aged 60 Years and Above, Excluding Unexposed Patients Vaccinated in the 3 Months Prior to the Start of the Campaign, VEBIS Primary Care Study, Europe **eTable 5.** Effectiveness of COVID-19 Vaccines Administered in the Autumn/Winter 2022/23 Against Infection With SARS-CoV-2 Variants Circulating Between September 2022 and August 2023, Among Adults Aged 60 Years and Above, Excluding Unexposed Patients Vaccinated in the 3 Months Prior to Symptom Onset, VEBIS Primary Care Study, Europe **eTable 6.** Effectiveness of COVID-19 Vaccines Administered in the Autumn/Winter 2022/23 Against Infection With SARS-CoV-2 Variants Circulating Between September 2022 and August 2023, Among Adults Aged 60 Years and Above, Excluding Unexposed Patients Vaccinated in the 6 Months Prior to Symptom Onset, VEBIS Primary Care Study, Europe

Supplement 2.
Nonauthor Collaborators

Supplement 3.
Data Sharing Statement
