# Supplementary material for: COVID-19 Vaccine Effectiveness in Autumn and Winter 2022 to 2023 Among Older Europeans
Source: JAMA Netw Open. 2024 Jul 1;7(7):e2419258. doi: 10.1001/jamanetworkopen.2024.19258 (PMC11217869; doi:10.1001/jamanetworkopen.2024.19258)
Supplement: Supplement 1. — eTable 1. Study Site-Specific COVID-19 Case Definition, VEBIS Primary Care Study, Europe, September 2022–August 2023 eTable 2. Start Date of the Autumn/Winter 2022/23 COVID-19 Vaccination Campaign by Study Site, VEBIS Primary Care Study, Europe, September 2022–August 2023 eTable 3. Effectiveness of COVID-19 Vaccines Administered in the Autumn/Winter 2022/23 Against Infection With SARS-CoV-2 Variants Circulating Between September 2022 and August 2023, Among Adults Aged 60 Years and Above, VEBIS Primary Care Study, Europe eTable 4. Effectiveness of COVID-19 Vaccines Administered in the Autumn/Winter 2022/23 Against Infection With SARS-CoV-2 Variants Circulating Between September 2022 and August 2023, Among Adults Aged 60 Years and Above, Excluding Unexposed Patients Vaccinated in the 3 Months Prior to the Start of the Campaign, VEBIS Primary Care Study, Europe eTable 5. Effectiveness of COVID-19 Vaccines Administered in the Autumn/Winter 2022/23 Against Infection With SARS-CoV-2 Variants Circulating Between September 2022 and August 2023, Among Adults Aged 60 Years and Above, Excluding Unexposed Patients Vaccinated in the 3 Months Prior to Symptom Onset, VEBIS Primary Care Study, Europe eTable 6. Effectiveness of COVID-19 Vaccines Administered in the Autumn/Winter 2022/23 Against Infection With SARS-CoV-2 Variants Circulating Between September 2022 and August 2023, Among Adults Aged 60 Years and Above, Excluding Unexposed Patients Vaccinated in the 6 Months Prior to Symptom Onset, VEBIS Primary Care Study, Europe [file jamanetwopen-e2419258-s001.pdf]

Supplemental Online Content

Laniece Delaunay C, Mazagatos C, Martínez-Baz I, et al; for the VEBIS Primary Care Vaccine Effectiveness Group. COVID-19 vaccine effectiveness in autumn and winter 2022 to 2023 among older Europeans. *JAMA Netw Open*. 2024;7(7):e2419258. doi:10.1001/jamanetworkopen.2024.19258

eTable 1. Study Site-Specific COVID-19 Case Definition, VEBIS Primary Care Study, Europe, September 2022–August 2023

eTable 2. Start Date of the Autumn/Winter 2022/23 COVID-19 Vaccination Campaign by Study Site, VEBIS Primary Care Study, Europe, September 2022–August 2023

eTable 3. Effectiveness of COVID-19 Vaccines Administered in the Autumn/Winter 2022/23 Against Infection With SARS-CoV-2 Variants Circulating Between September 2022 and August 2023, Among Adults Aged 60 Years and Above, VEBIS Primary Care Study, Europe

eTable 4. Effectiveness of COVID-19 Vaccines Administered in the Autumn/Winter 2022/23 Against Infection With SARS-CoV-2 Variants Circulating Between September 2022 and August 2023, Among Adults Aged 60 Years and Above, Excluding Unexposed Patients Vaccinated in the 3 Months Prior to the Start of the Campaign, VEBIS Primary Care Study, Europe

eTable 5. Effectiveness of COVID-19 Vaccines Administered in the Autumn/Winter 2022/23 Against Infection With SARS-CoV-2 Variants Circulating Between September 2022 and August 2023, Among Adults Aged 60 Years and Above, Excluding Unexposed Patients Vaccinated in the 3 Months Prior to Symptom Onset, VEBIS Primary Care Study, Europe

eTable 6. Effectiveness of COVID-19 Vaccines Administered in the Autumn/Winter 2022/23 Against Infection With SARS-CoV-2 Variants Circulating Between September 2022 and August 2023, Among Adults Aged 60 Years and Above, Excluding Unexposed Patients Vaccinated in the 6 Months Prior to Symptom Onset, VEBIS Primary Care Study, Europe

This supplemental material has been provided by the authors to give readers additional information about their work.

© 2024 Laniece Delaunay C et al. *JAMA Network Open*

eTable 1. Study site-specific COVID-19 case definition, VEBIS primary care study, Europe, September 2022–August 2023

Study site	COVID-19 case definition	COVID-19 vaccination information
Croatia	EU acute respiratory infection (ARI) case definition: sudden onset of symptoms and at least one of the four respiratory symptoms (cough, sore throat, shortness of breath or coryza) and a clinician's judgment that the illness is due to an infection	Depending on GP and region: Medical records, vaccination registry, GP interview (self-report)
France	Sudden onset of fever (or feverishness) and respiratory symptoms	GP interview (self-report)
Germany	At least one of the following symptoms: fever, cough, sore throat or nasal congestion	GP interview (self-report)
Hungary	EU ARI case definition	Medical records, GP interview (self-report)
Ireland	EU ARI case definition	Data linkage to vaccine registry
The Netherlands	EU ARI case definition	GP interview (self-report)
Portugal	EU ARI case definition	Vaccine registry look-up by GPs
Romania	EU ARI case definition	Medical records, GP interview (self-report)
Sweden	EU ARI case definition	Data linkage to vaccine registry as well as GP interview (self-report)
Spain, national	EU ARI case definition	GP interview (self-report)
Spain, Navarre region	EU influenza-like illness (ILI) case definition: sudden onset of symptoms and at least one of the four systemic symptoms (fever or feverishness, malaise, headache or myalgia) and at least one of the three respiratory symptoms (cough, sore throat, shortness of breath)	Data linkage to vaccine registry

eTable 2. Start date of the autumn/winter 2022/23 COVID-19 vaccination campaign by study site, VEBIS primary care study, Europe, September 2022–August 2023

Study site	Start date of the autumn/winter 2022/23 COVID-19 vaccination campaign
Croatia	September 12, 2022
France	October 3, 2022
Germany	October 6, 2022
Hungary	September 1, 2022
Ireland	September 15, 2022
The Netherlands	September 19, 2022
Portugal	September 7, 2022
Romania	November 25, 2022
Sweden	September 5, 2022
Spain, national	September 26, 2022
Spain, Navarre region	September 26, 2022

eTable 3. Effectiveness of COVID-19 vaccines administered in the autumn/winter 2022/23 against infection with SARS-CoV-2 variants circulating between September 2022 and August 2023, among adults aged 60 years and above, VEBIS primary care study, Europe

Estimate	Time since vaccination (days)	CVE (95% CI) (%)	N	Cases vacc	Cases unvacc	Controls vacc	Controls unvacc
September 2022–August 2023							
**Seasonal**	14–89	29 (14 to 42)	6,290	168	941	1,381	3,800
	90–179	16 (-4 to 33)	6,045	174	941	1,130	3,800
	180–269	-17 (-49 to 8)	5,442	187	941	514	3,800
	270–359	-73 (-171 to -10)	4,879	72	941	66	3,800
**Absolute**	14–89	39 (6 to 60)	1,867	168	82	1,381	236
	90–179	26 (-16 to 52)	1,622	174	82	1,130	236
	180–269	-84 (-245 to -3)	1,019	187	82	514	236
	270–359	NA	456	72	82	66	236
**Relative**	14–89	31 (15 to 44)	5,927	168	846	1,381	3,532
	90–179	16 (-4 to 33)	5,682	174	846	1,381	3,532
	180–269	-17 (-51 to 9)	5,079	187	846	514	3,532
	270–359	-63 (-160 to -3)	4,516	72	846	66	3,532
**Relative (2nd booster)**	14–89	34 (18 to 47)	4,946	143	700	1,200	2,903
	90–179	19 (-3 to 37)	4,763	150	700	1,010	2,903
	180–269	-14 (-48 to 13)	4,261	178	700	480	2,903
	270–359	-46 (-141 to 11)	3,738	71	700	64	2,903
XBB-dominant							
**Seasonal**	14–89	44 (-10 to 75)	1,309	9	268	81	951
	90–179	23 (0 to 41)	2,065	133	268	713	951
	180–269	-23 (-59 to 5)	1,913	186	268	508	951
	270–359	-91 (-207 to -20)	1,357	72	268	66	951
**Absolute**	14–89	52 (-23 to 82)	188	9	25	76	78
	90–179	39 (-11 to 65)	928	129	25	696	78
	180–269	-59 (-199 to 12)	785	183	25	499	78
	270–359	NA	240	72	25	65	78
**Relative**	14–89	47 (-8 to 77)	1,165	8	233	81	843
	90–179	24 (0 to 42)	1,905	128	233	701	843
	180–269	-22 (-59 to 6)	1,758	184	233	498	843
	270–359	-82 (-196 to -13)	1,213	72	233	65	843
**Relative (2nd booster)**	14–89	46 (-13 to 77)	906	8	186	72	640
	90–179	25 (0 to 45)	1,588	117	186	645	640
	180–269	-20 (-59 to 10)	1,469	174	186	469	640
	270–359	-69 (-185 to 0)	961	71	186	64	640

CI: confidence interval; CVE: COVID-19 vaccine effectiveness; unvacc: unvaccinated; vacc: vaccinated. All models are adjusted by age, sex, onset date, presence of commonly collected chronic conditions and study site.

© 2024 Laniece Delaunay C et al. *JAMA Network Open*

eTable 4. Effectiveness of COVID-19 vaccines administered in the autumn/winter 2022/23 against infection with SARS-CoV-2 variants circulating between September 2022 and August 2023, among adults aged 60 years and above, excluding unexposed patients vaccinated in the 3 months prior to the start of the campaign, VEBIS primary care study, Europe

Estimate	Time since vaccination (days)	CVE (95% CI) (%)	N	Cases vacc	Cases unvacc	Controls vacc	Controls unvacc
September 2022–August 2023							
**Seasonal**	14–89	27 (11 to 40)	6,487	168	967	1,381	3,971
	90–179	15 (-5 to 32)	6,242	174	967	1,130	3,971
	180–269	-16 (-48 to 9)	5,639	187	967	514	3,971
	270–359	-76 (-175 to -12)	5,076	72	967	66	3,971
**Relative**	14–89	28 (12 to 42)	6,124	168	872	1,381	3,703
	90–179	15 (-6 to 32)	5,879	174	872	1,130	3,703
	180–269	-16 (-49 to 9)	5,276	187	872	514	3,703
	270–359	-66 (-163 to -5)	4,713	72	872	66	3,703
**Relative (2nd booster)**	14–89	32 (15 to 45)	5,061	149	713	1,228	2,971
	90–179	21 (-1 to 38)	4,859	151	713	1,024	2,971
	180–269	-10 (-44 to 15)	4,349	179	713	486	2,971
	270–359	-43 (-135 to 13)	3,819	71	713	64	2,971
XBB-dominant							
**Seasonal**	14–89	46 (-7 to 75)	1,355	9	278	81	987
	90–179	22 (-1 to 40)	2,111	133	278	713	987
	180–269	-22 (-57 to 5)	1,959	186	278	508	987
	270–359	-92 (-208 to -21)	1,403	72	278	66	987
**Relative**	14–89	41 (-18 to 74)	1,220	9	244	81	886
	90–179	22 (-3 to 40)	1,976	133	244	713	886
	180–269	-22 (-59 to 6)	1,824	186	244	508	886
	270–359	-80 (-191 to -12)	1,268	72	244	66	886
**Relative (2nd booster)**	14–89	44 (-16 to 76)	921	8	192	72	649
	90–179	26 (0 to 44)	1,603	117	192	645	649
	180–269	-17 (-55 to 12)	1,484	174	192	469	649
	270–359	-65 (-178 to 2)	976	71	192	64	649

CI: confidence interval; CVE: COVID-19 vaccine effectiveness; unvacc: unvaccinated; vacc: vaccinated. All models are adjusted by age, sex, onset date, presence of commonly collected chronic conditions and study site.

© 2024 Laniece Delaunay C et al. *JAMA Network Open*

eTable 5. Effectiveness of COVID-19 vaccines administered in the autumn/winter 2022/23 against infection with SARS-CoV-2 variants circulating between September 2022 and August 2023, among adults aged 60 years and above, excluding unexposed patients vaccinated in the 3 months prior to symptom onset, VEBIS primary care study, Europe

Estimate	Time since vaccination (days)	CVE (95% CI) (%)	N	Cases vacc	Cases unvacc	Controls vacc	Controls unvacc
September 2022–August 2023							
**Seasonal**	14–89	26 (10 to 39)	6,657	168	988	1,381	4,120
	90–179	15 (-6 to 31)	6,412	174	988	1,130	4,120
	180–269	-16 (-48 to 8)	5,809	187	988	514	4,120
	270–359	-74 (-172 to -12)	5,246	72	988	66	4,120
**Relative**	14–89	27 (11 to 40)	6,294	168	893	1,381	3,852
	90–179	14 (-6 to 31)	6,049	174	893	1,130	3,852
	180–269	-16 (-48 to 9)	5,446	187	893	514	3,852
	270–359	-65 (-160 to -4)	4,883	72	893	66	3,852
**Relative (2nd booster)**	14–89	31 (15 to 45)	5,102	149	721	1,228	3,004
	90–179	21 (0 to 38)	4,900	151	721	1,024	3,004
	180–269	-9 (-41 to 16)	4,390	179	721	486	3,004
	270–359	-40 (-129 to 15)	3,860	71	721	64	3,004
XBB-dominant							
**Seasonal**	14–89	44 (-10 to 75)	1,401	9	287	81	1,024
	90–179	22 (-1 to 40)	2,157	133	287	713	1,024
	180–269	-23 (-59 to 4)	2,005	186	287	508	1,024
	270–359	-93 (-208 to -21)	1,449	72	287	66	1,024
**Relative**	14–89	41 (-16 to 74)	1,266	9	253	81	923
	90–179	21 (-3 to 40)	2,022	133	253	713	923
	180–269	-24 (-61 to 4)	1,870	186	253	508	923
	270–359	-82 (-193 to -13)	1,314	72	253	66	923
**Relative (2nd booster)**	14–89	46 (-12 to 77)	931	8	197	72	654
	90–179	27 (2 to 46)	1,613	117	197	645	654
	180–269	-16 (-53 to 12)	1,494	174	197	469	654
	270–359	-60 (-169 to 4)	986	71	197	64	654

CI: confidence interval; CVE: COVID-19 vaccine effectiveness; unvacc: unvaccinated; vacc: vaccinated. All models are adjusted by age, sex, onset date, presence of commonly collected chronic conditions and study site.

© 2024 Laniece Delaunay C et al. *JAMA Network Open*

eTable 6. Effectiveness of COVID-19 vaccines administered in the autumn/winter 2022/23 against infection with SARS-CoV-2 variants circulating between September 2022 and August 2023, among adults aged 60 years and above, <u>excluding unexposed patients vaccinated in the 6 months prior to symptom onset</u>, VEBIS primary care study, Europe

Estimate	Time since vaccination (days)	CVE (95% CI) (%)	N	Cases vacc	Cases unvacc	Controls vacc	Controls unvacc
September 2022–August 2023							
**Seasonal**	14–89	27 (11 to 40)	6,518	168	970	1,381	3,999
	90–179	15 (-5 to 32)	6,273	174	970	1,130	3,999
	180–269	-16 (-48 to 8)	5,670	187	970	514	3,999
	270–359	-74 (-172 to -12)	5,107	72	970	66	3,999
**Relative**	14–89	28 (12 to 41)	6,155	168	875	1,381	3,731
	90–179	15 (-6 to 32)	5,910	174	875	1,130	3,731
	180–269	-16 (-49 to 9)	5,307	187	875	514	3,731
	270–359	-64 (-160 to -4)	4,744	72	875	66	3,731
**Relative (2nd booster)**	14–89	32 (15 to 45)	5,067	149	715	1,228	2,975
	90–179	22 (0 to 38)	4,865	151	715	1,024	2,975
	180–269	-8 (-41 to 17)	4,355	179	715	486	2,975
	270–359	-39 (-128 to 15)	3,825	71	715	64	2,975
XBB-dominant							
**Seasonal**	14–89	44 (-10 to 75)	1,396	9	286	81	1,020
	90–179	22 (-1 to 40)	2,152	133	286	713	1,020
	180–269	-23 (-59 to 4)	2,000	186	286	508	1,020
	270–359	-92 (-207 to -21)	1,444	72	286	66	1,020
**Relative**	14–89	41 (-17 to 74)	1,261	9	252	81	919
	90–179	21 (-3 to 40)	2,017	133	252	713	919
	180–269	-24 (-61 to 4)	1,865	186	252	508	919
	270–359	-81 (-192 to -12)	1,309	72	252	66	919
**Relative (2nd booster)**	14–89	46 (-12 to 77)	931	8	197	72	654
	90–179	27 (2 to 46)	1,613	117	197	645	654
	180–269	-16 (-53 to 12)	1,494	174	197	469	654
	270–359	-60 (-169 to 4)	986	71	197	64	654

CI: confidence interval; CVE: COVID-19 vaccine effectiveness; unvacc: unvaccinated; vacc: vaccinated. All models are adjusted by age, sex, onset date, presence of commonly collected chronic conditions and study site.

© 2024 Laniece Delaunay C et al. *JAMA Network Open*
