# Supplementary material for: COVID-19 Vaccine Effectiveness in Autumn and Winter 2022 to 2023 Among Older Europeans
Source: JAMA Netw Open. 2024 Jul 1;7(7):e2419258. doi: 10.1001/jamanetworkopen.2024.19258 (PMC11217869; doi:10.1001/jamanetworkopen.2024.19258)
Supplement: Supplement 2. — Nonauthor Collaborators [file jamanetwopen-e2419258-s002.pdf]

Supplemental Online Content: Nonauthor Collaborators
*First name, last name, and suffix (if applicable) are required and will appear in PubMed.

*Group Name(s): VEBIS Primary Care Vaccine Effectiveness Group							
***First Name and Middle Initial(s)**	***Last Name**	***Suffix (eg, Jr, III)**	Academic Degrees	Institution	Location (city, state/province, country)	Role or Contribution, eg, chair, principal investigator	Group (if more than 1 Group listed in the byline) and/or Subgroup (eg, Steering Committee)
Virtudes	Gallardo García			Servicio de Vigilancia y Salud Laboral, Dirección General de Salud Pública y Ordenación Farmacéutica, Consejería de Salud y Consumo, Andalucía	Spain		
Esteban	Perez Morilla			Servicio de Vigilancia y Salud Laboral, Dirección General de Salud Pública y Ordenación Farmacéutica, Consejería de Salud y Consumo, Andalucía	Spain		
Irene	Pedrosa Corral			Servicio de Microbiología, Hospital Universitario Virgen de las Nieves, Granada	Spain		
Miriam	García Vázquez			Vigilancia Epidemiológica, Dirección General de Salud Pública, Departamento de Sanidad, Gobierno de Aragón	Spain		
Ana	Milagro-Beamonte			Laboratorio de Microbiología, Hospital Universitario Miguel Servet, Zaragoza. IIS Aragón, Zaragoza	Spain		
Ana	Fernandez Ibañez			Consejeria de Salud-Principado de Asturias	Spain		
Mario	Margolles Martins			Consejeria de Salud-Principado de Asturias; Instituto Investigación Principado de Asturias (ISPA)	Spain		
Jaume	Giménez Duran			Servicio de epidemiologia de la DG de Salud Pública de Baleares	Spain		

Supplemental Online Content: Nonauthor Collaborators
*First name, last name, and suffix (if applicable) are required and will appear in PubMed.

*First Name and Middle Initial(s)	*Last Name	*Suffix (eg, Jr, III)	Academic Degrees	Institution	Location (city, state/province, country)	Role or Contribution, eg, chair, principal investigator	Group (if more than 1 Group listed in the byline) and/or Subgroup (eg, Steering Committee)
Bartolomé	Sastre Palou			Servicio de epidemiologia de la DG de Salud Pública de Baleares	Spain		
Carla	López Causapé			Servicio de Microbiología, Hospital Universitario Son Espases, Palma	Spain		
Luis Javier	Viloria Raymundo			Vigilancia Epidemiológica. Dirección General de Salud Pública, Cantabria	Spain		
Tomás	Vega Alonso			Dirección General de Salud Pública, Junta de Castilla y León	Spain		
Ana	Ordax Díez			Dirección General de Salud Pública, Junta de Castilla y León	Spain		
Jose Eugenio	Lozano Alonso			Dirección General de Salud Pública, Junta de Castilla y León	Spain		
Silvia	Rojo Bello			Servicio de Microbiología, Hospital Clínico Universitario de Valladolid	Spain		
Jacobo	Mendioroz			Sub-Directorate General of Surveillance and Response to Public Health Emergencies, Public Health Agency of Catalonia, Generalitat of Catalonia, Barcelona	Spain		
Luca	Basile			Sub-Directorate General of Surveillance and Response to Public Health Emergencies, Public Health Agency of Catalonia, Generalitat of Catalonia, Barcelona	Spain		

Supplemental Online Content: Nonauthor Collaborators
*First name, last name, and suffix (if applicable) are required and will appear in PubMed.

***First Name and Middle Initial(s)**	***Last Name**	***Suffix (eg, Jr, III)**	Academic Degrees	Institution	Location (city, state/province, country)	Role or Contribution, eg, chair, principal investigator	Group (if more than 1 Group listed in the byline) and/or Subgroup (eg, Steering Committee)
Ana Isabel	Martínez Mateo			Sub-Directorate General of Surveillance and Response to Public Health Emergencies, Public Health Agency of Catalonia, Generalitat of Catalonia, Barcelona	Spain		
Carlota	Ruiz de Porras			Sub-Directorate General of Surveillance and Response to Public Health Emergencies, Public Health Agency of Catalonia, Generalitat of Catalonia, Barcelona	Spain		
Alba	Moya Garcés			Sub-Directorate General of Surveillance and Response to Public Health Emergencies, Public Health Agency of Catalonia, Generalitat of Catalonia, Barcelona	Spain		
Mª Ángeles	Marcos			Laboratorio de Microbiología, Hospital Clínic de Barcelona	Spain		
Aurora	López Maside			Subdirección General de Epidemiologia y Vigilancia de la Salud, Valencia	Spain		
Francesc	Botella Quijal			Subdirección General de Epidemiologia y Vigilancia de la Salud, Valencia	Spain		
Maite	Miralles Espi			Subdirección General de Epidemiologia y Vigilancia de la Salud, Valencia	Spain		

Supplemental Online Content: Nonauthor Collaborators
*First name, last name, and suffix (if applicable) are required and will appear in PubMed.

***First Name and Middle Initial(s)**	***Last Name**	***Suffix (eg, Jr, III)**	Academic Degrees	Institution	Location (city, state/province, country)	Role or Contribution, eg, chair, principal investigator	Group (if more than 1 Group listed in the byline) and/or Subgroup (eg, Steering Committee)
Cristina	Andreu Salete			Subdirección de Epidemiología. Dirección General de Salud Pública. Servicio Extremeño de Salud, Mérida	Spain		
María del Carmen	García Rodríguez			Subdirección de Epidemiología. Dirección General de Salud Pública. Servicio Extremeño de Salud, Mérida	Spain		
Juan Antonio	Linares			Subdirección de Epidemiología. Dirección General de Salud Pública. Servicio Extremeño de Salud, Mérida	Spain		
Luis	García Comas			Subdirección General de Vigilancia en Salud Pública, Madrid	Spain		
Mª Isabel	Barranco			Servicio de Epidemiología, Sección de Vigilancia Epidemiológica, Consejería de Salud de Murcia	Spain		
María-Dolores	Chirlaque			Servicio de Epidemiología, Sección de Vigilancia Epidemiológica, Consejería de Salud de Murcia	Spain		
Antonio	Moreno Docón			Servicio de Microbiología, Hospital Clínico Universitario Virgen de Arrixaca	Spain		
Violeta	Ramos Marín			Servicio de Vigilancia Epidemiológica de la Consejería de Sanidad y Servicios Sociales de Ceuta	Spain		

*First name, last name, and suffix (if applicable) are required and will appear in PubMed.

***First Name and Middle Initial(s)**	***Last Name**	***Suffix (eg, Jr, III)**	Academic Degrees	Institution	Location (city, state/province, country)	Role or Contribution, eg, chair, principal investigator	Group (if more than 1 Group listed in the byline) and/or Subgroup (eg, Steering Committee)
Daniel	Castrillejo			Servicio de Epidemiología, Dirección General de Salud Pública, Consejería de Políticas Sociales y Salud Pública de Melilla	Spain		
Atanasio	Gómez Anés			Servicio de Epidemiología, Dirección General de Salud Pública, Consejería de Políticas Sociales y Salud Pública de Melilla	Spain		
Amparo	Larrauro			Centro Nacional de Epidemiología, Instituto de Salud Carlos III, Madrid	Spain		
Gloria	Pérez-Gimeno			Centro Nacional de Epidemiología, Instituto de Salud Carlos III, Madrid	Spain		
Marcos	Lozano Álvarez			Centro Nacional de Epidemiología, Instituto de Salud Carlos III, Madrid	Spain		
Lorena	Vega			Centro Nacional de Epidemiología, Instituto de Salud Carlos III, Madrid	Spain		
Silvia	Galindo			Centro Nacional de Epidemiología, Instituto de Salud Carlos III, Madrid	Spain		
Tania	Puma			Centro Nacional de Epidemiología, Instituto de Salud Carlos III, Madrid	Spain		
Susana	Monge			Centro Nacional de Epidemiología, Instituto de Salud Carlos III, Madrid	Spain		

Supplemental Online Content: Nonauthor Collaborators
*First name, last name, and suffix (if applicable) are required and will appear in PubMed.

***First Name and Middle Initial(s)**	***Last Name**	***Suffix (eg, Jr, III)**	Academic Degrees	Institution	Location (city, state/province, country)	Role or Contribution, eg, chair, principal investigator	Group (if more than 1 Group listed in the byline) and/or Subgroup (eg, Steering Committee)
Francisco	Pozo			Centro Nacional de Microbiología, Instituto de Salud Carlos III, Madrid	Spain		
Inmaculada	Casas			Centro Nacional de Microbiología, Instituto de Salud Carlos III, Madrid	Spain		
Virginia	Sandonis			Centro Nacional de Microbiología, Instituto de Salud Carlos III, Madrid	Spain		
Sonia	Vázquez-Morón			Centro Nacional de Microbiología, Instituto de Salud Carlos III, Madrid	Spain		
Aitziber	Echeverría		PhD	Instituto de Salud Pública de Navarra - IdiSNA - CIBERESP	Navarre, Spain		
Camino	Trobajo-Sanmartín		PhD	Instituto de Salud Pública de Navarra - IdiSNA - CIBERESP	Navarre, Spain		
Manuel	García Cenoz		MD, PhD	Instituto de Salud Pública de Navarra - IdiSNA - CIBERESP	Navarre, Spain		
Guillermo	Ezpeleta		MD	Instituto de Salud Pública de Navarra - IdiSNA - CIBERESP	Navarre, Spain		
Carmen	Ezpeleta		MD	Hospital Universitario de Navarra - IdiSNA	Navarre, Spain		
Ana	Navascués		MD	Hospital Universitario de Navarra - IdiSNA	Navarre, Spain		
Katalin	Krisztalovics			Semmelweis University	Hungary		
Krisztina	Mucsányiné Juhász			Semmelweis University	Hungary		
Katalin	Kristóf			Semmelweis University	Hungary		
Ute	Preuss			Robert Koch Institut	Germany		
Marianne	Wedde			Robert Koch Institut	Germany		
Barbara	Biere			Robert Koch Institut	Germany		
Janine	Reiche			Robert Koch Institut	Germany		
Djin-Ye	Oh			Robert Koch Institut	Germany		
Adele	McKenna			Health Protection Surveillance Centre	Ireland		

Supplemental Online Content: Nonauthor Collaborators
*First name, last name, and suffix (if applicable) are required and will appear in PubMed.

*First Name and Middle Initial(s)	*Last Name	*Suffix (eg, Jr, III)	Academic Degrees	Institution	Location (city, state/province, country)	Role or Contribution, eg, chair, principal investigator	Group (if more than 1 Group listed in the byline) and/or Subgroup (eg, Steering Committee)
Jeff	Connell			University College Dublin	Ireland		
Michael	Joyce				Ireland		
Mariam	Bagheri			National Institute for Public Health and the Environment (RIVM)	Netherlands		
Sanne	Bos			National Institute for Public Health and the Environment (RIVM)	Netherlands		
Sharon	van den Brink			National Institute for Public Health and the Environment (RIVM)	Netherlands		
Frederika	Dijkstra			National Institute for Public Health and the Environment (RIVM)	Netherlands		
Dirk	Eggink			National Institute for Public Health and the Environment (RIVM)	Netherlands		
Rianne	van Gageldonk-Lafeber			the Environment (RIVM)	Netherlands		
Gabriel	Goderski			National Institute for Public Health and the Environment (RIVM)	Netherlands		
Chantal	Herrebrugh			National Institute for Public Health and the Environment (RIVM)	Netherlands		
Liz	Jenniskens			National Institute for Public Health and the Environment (RIVM)	Netherlands		
Daphne	Reukers			National Institute for Public Health and the Environment (RIVM)	Netherlands		
John	Sluimer			National Institute for Public Health and the Environment (RIVM)	Netherlands		
Tara	Sprong			National Institute for Public Health and the Environment (RIVM)	Netherlands		
Anne	Teirlinck			National Institute for Public Health and the Environment (RIVM)	Netherlands		
Nienke	Veldhijzen			Nivel Netherlands Institute for Health Services Research	Netherlands		
Ruben	van der Burgh			Nivel Netherlands Institute for Health Services Research	Netherlands		
Cathrien	Kager			Nivel Netherlands Institute for Health Services Research	Netherlands		
Mayra	Klinkhamer			Nivel Netherlands Institute for Health Services Research	Netherlands		
Bart	Knottnerus			Nivel Netherlands Institute for Health Services Research	Netherlands		
Marloes	Riethof			Nivel Netherlands Institute for Health Services Research	Netherlands		

*First name, last name, and suffix (if applicable) are required and will appear in PubMed.

***First Name and Middle Initial(s)**	***Last Name**	***Suffix (eg, Jr, III)**	Academic Degrees	Institution	Location (city, state/province, country)	Role or Contribution, eg, chair, principal investigator	Group (if more than 1 Group listed in the byline) and/or Subgroup (eg, Steering Committee)
Ruud	van den Broek			Nivel Netherlands Institute for Health Services Research	Netherlands		
Safira	Wortel			Nivel Netherlands Institute for Health Services Research	Netherlands		
Ausenda	Machado			National Institute of Health Doutor Ricardo Jorge	Portugal		
Irina	Kislaya			National Institute of Health Doutor Ricardo Jorge	Portugal		
Carlos	Aniceto			National Institute of Health Doutor Ricardo Jorge	Portugal		
Licínia	Gomes			National Institute of Health Doutor Ricardo Jorge	Portugal		
Nuno	Verdasca			National Institute of Health Doutor Ricardo Jorge	Portugal		
Camila	Henriques			National Institute of Health Doutor Ricardo Jorge	Portugal		
Daniela	Dias			National Institute of Health Doutor Ricardo Jorge	Portugal		
Miguel	Lança			National Institute of Health Doutor Ricardo Jorge	Portugal		
Thierry	Blanchon				France		
Caroline	Guerrisi				France		
Aubane	Renard				France		
Titouan	Launay				France		
Shirley	Masse				France		
Marie	Chazelle				France		
Ivana	Ferenčak				Croatia		
Bernard	Kaić				Croatia		
Vesna	Višekruna Vučina				Croatia		
Katica	Čusek Adamić				Croatia		
Mirjana Lana	Kosanović Ličina				Croatia		
Danijela	Lakošeljac				Croatia		
Ivana	Mihin Huskić				Croatia		
Diana	Nonković				Croatia		

Supplemental Online Content: Nonauthor Collaborators
*First name, last name, and suffix (if applicable) are required and will appear in PubMed.

***First Name and Middle Initial(s)**	***Last Name**	***Suffix (eg, Jr, III)**	Academic Degrees	Institution	Location (city, state/province, country)	Role or Contribution, eg, chair, principal investigator	Group (if more than 1 Group listed in the byline) and/or Subgroup (eg, Steering Committee)
Annasara	Carnahan				Sweden		
Eva	Hansson-Pihlainen				Sweden		
Elin	Arvesen				Sweden		
Nora	Nid				Sweden		
Anna-Lena	Hansen				Sweden		
Emmi	Andersson				Sweden		
Lena	Dillner				Sweden		
Adrian	Jidovu				Romania		
Olivia Carmen	Timnea				Romania		
Cătălina	Pascu				Romania		
Mihaela	Oprea				Romania		
Iulia	Bistriceanu				Romania		
Alina	Ivanciuc				Romania		
Maria Elena	Mihai				Romania		
