# Supplementary material for: COVID-19 Vaccine Effectiveness in Autumn and Winter 2022 to 2023 Among Older Europeans
Source: JAMA Netw Open. 2024 Jul 1;7(7):e2419258. doi: 10.1001/jamanetworkopen.2024.19258 (PMC11217869; doi:10.1001/jamanetworkopen.2024.19258)
Supplement: Supplement 3. — Data Sharing Statement [file jamanetwopen-e2419258-s003.pdf]

Data Sharing Statement

Laniece Delaunay. COVID-19 Vaccine Effectiveness in Autumn and Winter 2022 to 2023 Among Older Europeans. *JAMA Netw Open*. Published July 01, 2024. doi:10.1001/jamanetworkopen.2024.19258

Data
Data available: Yes
Data types: Other (please specify)
Additional Information: Aggregate data
How to access data: Aggregate data available on request (e.kissling@epiconcept.fr).
When available: With publication

Supporting Documents
Document types: None

Additional Information
Who can access the data: Researchers whose proposed use of the data has been approved
Types of analyses: For public health purposes.
Mechanisms of data availability: After approval of a proposal
